# Genetic distinctiveness of an endangered falcon: Implications for conservation in Europe

**DOI:** 10.1371/journal.pone.0295424

**Published:** 2023-12-20

**Authors:** Lorenzo Attili, Luisa Garofalo, Giuseppe Puddu, Giampiero Tirone, Antonella Pizzarelli, Nicholas Barbara, Elisabeth Haring, Rita Lorenzini

**Affiliations:** 1 Istituto Zooprofilattico Sperimentale del Lazio e della Toscana *M*. *Aleandri*, Centro di Referenza Nazionale per la Medicina Forense Veterinaria, Grosseto, Italy; 2 Department of Biology and Biotechnology “*C*. *Darwin*”, Sapienza University of Rome, Rome, Italy; 3 Istituto Zooprofilattico Sperimentale del Lazio e della Toscana *M*. *Aleandri*, Rome, Italy; 4 Regione Lazio, Riserva Naturale Lago di Vico, Caprarola, Viterbo, Italy; 5 Birdlife Malta, Ta’ Xbiex, Malta; 6 Natural History Museum Vienna, Wien, Austria; Chang Gung University, TAIWAN

## Abstract

In the Falconidae, the genus *Falco* comprises species of large birds of prey with wide distribution worldwide. However, the European lanner falcon *Falco biarmicus feldeggii* is rapidly heading for global extinction following a dramatic decline caused by anthropogenic interference. Conservation projects are currently underway with the main purpose of increasing its population size in the Mediterranean basin through captive breeding and release of birds into the wild. To support the projects, and strengthen the legitimacy of conservation efforts consistently with the Evolutionary Significant Unit concept, we explored the possibility of characterising the gene pool of the European lanner and reliably distinguishing it from other falcon taxa inhabiting the Mediterranean area, which show morphological and genetic similarities. To address the issue, we examined genetic variability at the nuclear level through the analysis of 12 neutral Short Tandem Repeat loci, and, for the first time in these taxa, two single-copy functional genes, coding for the brain-derived neurotrophic factor precursor and the oocyte maturation factor, respectively. The second exon of the *major histocompatibility complex class II B* gene was also investigated. Additionally, to frame our data with previously published data, we assess variation at the mitochondrial level by sequencing portions of the *cytochrome b*, *12S* rRNA gene, and the control region. Our results showed that the European lanner is highly distinct from other falcon taxa, as revealed by nuclear, but not by mitochondrial DNA. We discuss our findings focusing on their implications for the preservation of this highly endangered European bird, and highlighted the critical role of genetic information in planning and monitoring concrete interventions.

## Introduction

In the hierofalcon complex (Aves: Falconidae), the genus *Falco* comprises five species of large birds of prey (saker falcon *Falco cherrug*, lanner falcon *Falco biarmicus*, gyrfalcon *Falco rusticolus* and laggar falcon *Falco jugger*) with wide distribution in the Palearctic, Nearctic and Afrotropical regions. However, many falcon populations are experiencing intense decline in different areas of the range due to direct and accidental human interference ([1] and references therein). While inhabiting very diverse habitats, these raptors show high morphological similarity and surprisingly poor genetic differentiation at the mitochondrial level, possibly as a consequence of their recent radiation and interspecific gene flow through hybridisation [1–3].

**Fig 1:**
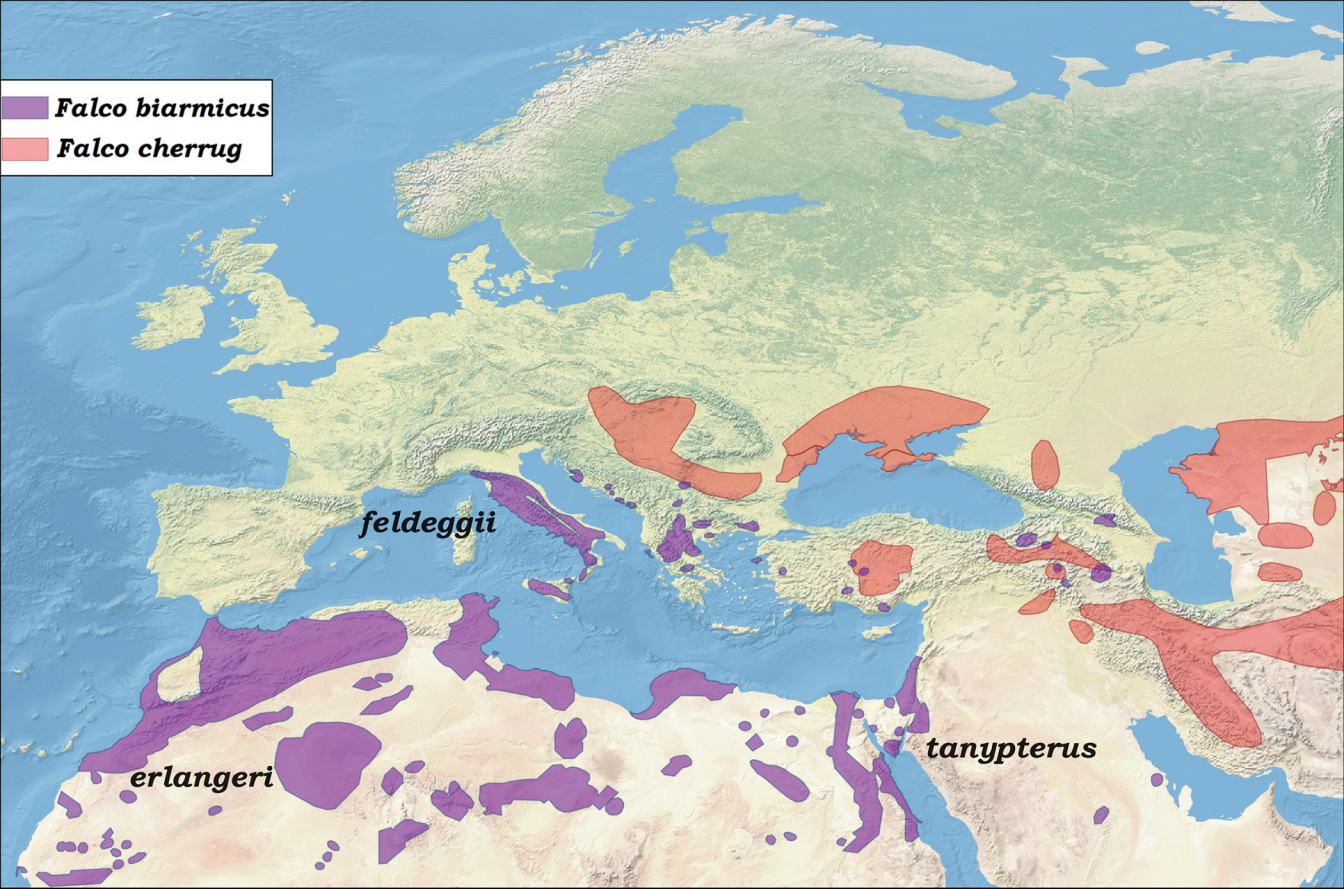
Distribution of *F*. *biarmicus* and *F*. *cherrug* in the Mediterranean area. Only areas where the species occur as resident and breeding native are indicated (redrawn from BirdLife International 2022, public domain, web site http://datazone.birdlife.org/species/requestdis). The individual ranges for subspecies of *F*. *biarmicus* are not indicated because it is not clear exactly which areas they cover and where they meet. Map layout was freely available online from Natural Earth at https://www.naturalearthdata.com/.

The polytypic lanner falcon *F*. *biarmicus* is widespread in the Mediterranean area with three putative subspecies: *F*. *biarmicus feldeggii* dwelling in Italy, the Balkans, Greece, Turkey and the southern Caucasus areas, *F*. *biarmicus erlangeri* in north western Africa, and *F*. *biarmicus tanypterus* occurring from northeastern Africa eastward into Israel (Fig 1). Whether *tanypterus* and *erlangeri* are different taxonomic entities, or rather are the same subspecies, is a matter of debate [4, 5]. The saker falcon *F*. *cherrug*, which reaches as far as the eastern Alps as the westernmost limit of its range, is the hierofalcon species that is geographically closest to *F*. *biarmicus* in the Mediterranean area, where it probably meets the European lanner falcon *F*. *b*. *feldeggii*. The latter is highly threatened with extinction over the entire range, with no more than 200 breeding pairs worldwide [6]. In peninsular Italy and Sicily, in particular, only 140–172 pairs still remained a few years ago [7], while in central Italy the population decreased from 10–15 pairs in the 1980s, to no breeding pairs at all in 2016 [6]. The European lanner is currently listed in the Birds Directive 2009/147/CE, Appendix II of the Bonn Convention, Annex II of the Bern Convention, and Appendix II of the Convention on International Trade in Endangered Species of Wild Fauna and Flora (CITES). Furthermore, it is categorised as “near threatened” by the International Union for Conservation of Nature’s (IUCN) Red List of Threatened Species (https://www.iucn.org), and classified as a European Species of Conservation Concern (SPEC level 3).

*Falco b*. *feldeggii* has been poorly investigated genetically, both in terms of number of individuals, populations and genome coverage [1, 3, 8, 9]. Most of previous molecular studies in the hierofalcon complex were based on variation at the mitochondrial (mt) DNA. Partial mt sequences of the *cytochrome b* gene (*cytB*) and the non-coding control region (CR) showed that the extant mitochondrial lineages did not identify any *Falco* species or subspecies (*F*. *b*. *feldeggii* included) as a monophyletic group. Therefore, these molecular markers proved to be of little diagnostic utility for (sub)species identification, nor were they in accordance with current taxonomy [3]. The occurrence of interbreeding between hierofalcon species, both natural and human-mediated through the practice of falconry, further complicates their differentiation [1, 3].

At the genomic level, some species of Falconidae have been investigated through the analysis of the major histocompatibility complex, MHC [8, 10–12]. *MHC* genes have recently gained much attention in evolutionary studies and conservation biology in birds of prey due to their exceptionally high heterozygosity compared to neutral markers [13]. However, only the saker falcon and the kestrels (*Falco naumanni* and *Falco tinnunculus*) have been extensively examined across their Eurasian ranges, actually revealing high levels of polymorphism [11, 12]. On the contrary, few individuals have been analysed for other falcons, including *F*. *biarmicus*. These preliminary results, however, revealed surprisingly low ancestral intra- and interspecific MHC variability [8].

In order to strengthen the legitimacy of conservation efforts (*sensu* Moritz [14]), and to proactively support ongoing projects for the reintroduction/restocking of the European lanner (LIFE ConRaSI: https://www.lifeconrasi.eu/; LIFE LANNER: https://www.lifelanner.eu/it/), we explored the possibility of characterising the gene pool of *F*. *b*. *feldeggii* and reliably distinguishing it from the other subspecies inhabiting the Mediterranean area (*erlangeri* and *tanypterus*) and their possible crossbreds. It is worth mentioning, however, that this goal is far from trivial, since earlier genetic studies indicated very little differentiation for the Palearctic taxa of *F*. *biarmicus*, so as to rekindle the debate on their subspecific designation ([9] and references therein).

Additionally, we aimed at differentiating *F*. *b*. *feldeggii* from the saker falcon *F*. *cherrug*, which is geographically the closest species, and therefore potential candidate for hybridisation. To address the issue, we first examined genetic variability at the nuclear level in *F*. *biarmicus* ssp. and *F*. *cherrug* through the analysis of 12 Short Tandem Repeat (STR) loci, selected as neutral genetic markers. Secondly, we analysed variation at portions of two single-copy functional genes, previously used for phylogeny studies in birds [15], coding for the brain-derived neurotrophic factor precursor (BDNF) and the oocyte maturation factor (Cmos). They are studied here for the first time in these taxa. Furthermore, we investigated variability at the MHC class II B gene, including more falcon taxa and a higher number of individuals than in previous studies [8, 10, 12]. The study of neutral loci and functional genes under selective pressure provides a more comprehensive picture of genetic variability [16].

Genetic variation was also assessed at the mitochondrial level by sequencing portions of the *cytB* gene, the *12S* rRNA gene (*12S*), and the CR, primarily to frame our data in a context of greater variability, through comparisons with the available published results from other hierofalcon taxa and multiple geographic areas.

Finally, we discuss our genetic findings focusing on their implications for the conservation of the highly endangered European lanner, highlighting the critical role of genetic information in planning and monitoring concrete interventions.

## Materials and methods

### Sample collection and DNA extraction

A total of 115 samples of lanner *F*. *biarmicus* ssp. and saker falcon *F*. *cherrug* were gathered from different Mediterranean countries, museums and collections (Table 1, S1 Table). Samples consisted of plucked feathers and blood from live lanners held in captivity because sick, injured in the wild, seized by the authorities, or because involved in LIFE LANNER Project. Skin and feathers of taxidermied falcons were obtained from European collections. Most stuffed specimens were recent (N = 38), dating from no earlier than 1950, while some of them (N = 7) dated from 1847 to 1953. In addition, fresh muscle tissues were opportunistically collected from individuals that died naturally, accidentally or killed illegally. No birds were wild-caught, nor were any live birds handled for the purpose of this study, rather only as part of conservation actions and health interventions under regular permissions from the countries of origin. All necessary permits were obtained for the described study, which complied with all relevant CITES regulations.

**Table 1:** Hierofalcon samples analysed in this study, and corresponding concatenated and control-region-based (CR) mitochondrial haplotypes.

Taxon	Geographic area	Field	Museum	Concatenated haplotype	CR haplotype
*Falco biarmicus feldeggii* (N = 61)	Peninsular Italy	45	6^a^^,^^b^^,^^c^	H3 H6	H3 H6
	Sicily		1^a^	H6	H6
	Spain	8		H3 H6	H3 H6
	Dalmatia		1^c^	na	na
*F*. *b*. *tanypterus* (N = 14)	Israel	14		H7 H4	H3 H4
*F*. *b*. *erlangeri* (N = 14)	North Africa (unknown location)	3	4^d^	H7 H1 H2	H3 H1 H2
	Egypt		7^d^	H5 H3 H6	H5 H3 H6
*F*. *cherrug* (N = 26)	Austria		17^c^	H7 H3 H6	H3 H3 H6
	Slovakia		2^c^	H3	H3
	Unknown		7^c^	H8 H3 H6	H8 H3 H6
**N = 115**		**N = 70**	**N = 45**		

na = not amplified; N = sample size (see text for details).

^a^ Carmagnola Natural History Museum (Torino, Italy)

^b^ Museum of the University "G. D’Annunzio" (Chieti, Italy)

^c^ Natural History Museum of Vienna (Wien, Austria)

^d^ National Museum of Natural History (Mdina, Malta).

Total DNA was isolated from approximately 15 mg of muscle or 200 μl of whole blood in EDTA preserved at -20°C, using the QIAamp DNA Mini Kit (QIAGEN, Hilden, Germany). The DNA from feather calamus and skin of museum specimens was extracted with the Maxwell16 Instrument (Promega, Madison, USA) for automated genomic DNA isolation following the user handbook, with an overnight incubation in two-fold lysis buffer volume (containing 1M dithiothreitol) as minor adjustment. Extracted DNAs were diluted in 100 μl RNAse-free molecular grade water and quantified with the Quantus Fluorometer (Promega) according to the manufacturer’s instructions. One mock tube with reagents and no sample DNA was included in each extraction round. Museum samples were processed separately from fresh tissues using dedicated equipment in different extraction/amplification sessions.

### STR genotyping

We selected and amplified 18 STR loci that were isolated originally in *Falco peregrinus* (FP01, FP89, FP92-1, FP46-1, FP86-2, FP5, FP13, FP54, FP31, FP347, FP82-2, Fp79-1, FP79-4, FP107; [1, 17]), *Falco vespertinus* (FALVES-03, FALVES-31, FALVES-13; [18]), and *Accipiter gentilis* (AGE-5; [19]). Two loci (FP01, FP79-4) failed to amplify, and four (FP92-1, FP 46–1, FP86-2, FALVES-13) proved monomorphic in a pilot subset of twenty lanners. Consequently, six loci were excluded from further analyses. Amplifications of the remaining 12 loci were optimised in two multiplexes (Table 2). Each multiplex contained 2 μl of genomic DNA, 0.8 μM of each primer, 3.6 μl of master mix (QIAGEN Multiplex PCR Kit) and PCR grade H_2_O in 10.4 μl total reaction volume. Multiplex amplifications were performed in a Veriti thermal cycler (Applied Biosystems), and consisted of an initial 15 min activation step at 95°C, followed by 32 cycles of denaturation at 94°C for 30 sec, annealing at 55°C for 90 sec, extension at 72°C for 60 sec, and a final extension step at 60°C for 30 min. Extraction negative controls and PCR mock reactions were included in each amplification session. A volume of 2 μl of five-fold diluted amplified reaction products was denatured with 14 μl HiDi^TM^ formamide (Applied Biosystems), combined with 0.2 μl GeneScan^TM^ 500 LIZ^TM^ size standard (Applied Biosystems) and loaded on the ABI Prism^TM^ 3130 Genetic Analyzer. GeneMapper Software 5.0 was used for fragment sizing and allele calling.

**Table 2:** STR loci, fluorescent dye labels at 5’ of the forward primer, and allelic ranges in the genotyped falcon samples.

Locus	Dye label	Multiplex	Allelic range (bp)
AGE-5	PET	1	159–169
FP13	FAM	1	94–102
FP79-1	NED	1	140–146
FP107	FAM	1	191–209
FP54	PET	1	100–120
FP347	FAM	1	130–154
FALVES-03	FAM	2	159–189
FP82-2	NED	2	131–153
FALVES-31	FAM	2	119–153
FP31	PET	2	144–156
FP5	FAM	2	97–103
FP89	VIC	2	118–122

### Amplification and sequencing of nuclear coding genes and mitochondrial markers

Portions of the mitochondrial *cytB*, *12S*, CR, as well as fragments of the nuclear protein-coding genes *BDNF* and *Cmos*, and the second exon of the *MHC class II B* gene were amplified and sequenced using published primers and primers developed *de novo* for this study (Table 3). The algorithm in Primer3 (https://bioinfo.ut.ee/primer3-0.4.0/) was employed to design the new primers based on the reference mitogenome of *Falco cherrug* (deposited in GenBank https://www.ncbi.nlm.nih.gov/genbank/ with Accession number NC026715).

**Table 3:** Primers and amplification details for nuclear and mitochondrial amplicons.

Amplicon	Primer sequences 5′–3′	Length (bp)	T (°C)	Reference
12S	267F: AAAAACGTTAGGTCAAGG 267R: GTATGCTTACCTTGTTAC	225	52	This study
Cytb	ND5-mod-For: TATCTAGGATCTTTCGCCCT H15149: AAACTGCAGCCCCTCAGAATGATATTTGTCCTCA	445	55	[20] [21]
CR	LAN-CR-F: GGTGACCCTTCTGAGTGC LAN-CR-R: GGGTGTGAATTTTGGTGG	290	55	This study ^a^
MHC	Fal2FC: CCTCCCTGTACAAACAGAG Fal2RC: GTGGCACTGGGAAACSTG	265	56	[10]
BDNF	BDNF-F: GACCATCCTTTTCCTKACTATGGTTATTTCATACTT BDNF-R: CTATCTTCCCCTTTTAATGGTCAGTGTACAAAC	670	58	[22]
Cmos	Cmos1: GCCTGGTGCTCCATCGACTGGGATCA Cmos3: GTAGATGTCTGCTTTGGGGGTGA	590	58	[23]

T = temperature of annealing; bp = base pairs. ^a^ Primers were designed to avoid amplifications of numts, while covering the most variable CR portion (see [3]).

The same PCR reactions were used for all fragments and contained 2.5 μl of 10X Gold buffer (Applied Biosystems, USA), 200 μM of each dNTP, 2.5 mM MgCl_2_, 1.5 μM of each primer (Table 3), 1U of AmpliTaqGold polymerase (Applied Biosystems) and 3μl template DNA (approximately 20 ng) in a final volume of 25 μl. Amplifications consisted of an initial denaturation step at 94°C for 3 min, 38 cycles of 30 s at 94°C, 30 s at the annealing temperature (Table 3), 30 s extension step at 72°C for 30 s, followed by 5 min at 72°C. Extraction and PCR negative controls were included in each amplification round to monitor contaminations. Amplified products were visualised under UV light after electrophoresis on 1.5% agarose gel and staining with Gel Red^TM^ (Biotium, Inc., USA). A 50–2000 bp DNA ladder (Sigma-Aldrich Chemicals, Italy) was used to verify the molecular size of amplicons. PCR products were cleaned up with the QIAquick PCR purification kit (QIAGEN, Hilden, Germany) and sequenced bidirectionally using the amplification primers and the BigDye Terminator kit v3.1 (Applied Biosystems) according to the manufacturer’s protocol. Unincorporated dyes and other contaminants were removed with the Agencourt® CleanSEQ solution (Beckman Coulter, USA), then sequences were loaded on the ABI Prism^TM^ 3130 Genetic Analyzer and analysed using the Sequencing Analysis Software v5.3.1.

### Data analysis

#### Nuclear coding genes and STRs

After amplification and sequencing of *MHC*, *BDNF* and *Cmos* genes, the chromatograms were inspected and sequences without double peaks at any site (indicating homozygotes) were recorded as real alleles (or haplotypes) when their occurrence in the homozygous state was verified in at least three different individuals. When sequences exhibited double peaks, each polymorphic position was carefully scanned by eye to check for accuracy and consistency of double peaks across all heterozygous individuals. As an additional evidence for true polymorphic positions, we verified that double peaks in the chromatograms were lower than adjacent single peaks, as compared to homozygous individuals at that site. Haplotypes of heterozygotes at multiple sites were reconstructed *via* computation based on a Bayesian algorithm [24]. The computational approach was used as an efficient and cost-effective alternative method to standard cloning for the separation of the constituent alleles [25]. For this purpose, we employed the package PHASE v2.1.1, (https://stephenslab.uchicago.edu/phase/download.html) to phase the observed genotype data under the recombination model using the default settings [24]. Allelic states from unphased haplotypes were statistically inferred and the phase probability value at each variable site was derived. If the probability for the reconstructed alleles was less than 0.90, due to the possibility of multiple allele combinations or the presence of polymorphisms occurring only once in the data set, then the unresolved “orphan” alleles [25] were recorded as missing values. To check for the misamplification of duplicated or pseudogenes, the sequences of alleles from homozygous individuals and reconstructed alleles from heterozygotes were verified against *F*. *cherrug* reference genome (Accession number GCF_000337975.1). Additionally, the sequences were matched and aligned with those available in GenBank for the *Falco* (sub)species using the BLAST algorithm [26] (https://blast.ncbi.nlm.nih.gov).

Single genotypes were obtained at 12 STR loci and overall individual multilocus profiles based on a 15-loci complete panel, consisting of both neutral STRs and coding genes, were obtained for all samples. Departures from linkage equilibrium (LE) between loci were assessed in GENEPOP v4.7.5 [27] (https://genepop.curtin.edu.au) using 10^4^ steps of dememorisation, 10^3^ batches and 10^4^ iterations per batch. The software MICRO-CHECKER 2.2.3 [28] was used to evaluate potential genotyping errors and frequencies of null alleles at single loci.

A Discriminant Analysis of Principal Components, DAPC [29] based on multilocus profiles was performed using the Adegenet package 2.1.6 [30] for the R software v.4.2.1 on the web interface. This multivariate approach identifies discrete clusters of individuals, maximising variation among groups while minimising variation within groups, without relying on any underlying genetic model. The analysis allowed us to visualise and qualitatively describe genetic variation at the population level based on our entire falcon dataset. Clusters of samples were initially pre-defined according to their collection location/taxonomic status. The optimal number of principal components that were retained was determined by cross validation.

To complement the multivariate analysis of nuclear data, a Bayesian assignment procedure as implemented in the software STRUCTURE v.2.3.4 [31] was used to identify clusters (K) of genetically close individuals and admixed genotypes based on a Markov Chain of Monte Carlo with 5 x 10^6^ iterations and 5 x 10^5^ steps of burn-in to achieve convergence. Ten repetitions for each K ranging from 2 to 8 were performed under an admixture model using independent allele frequencies, without assuming any prior nongenetic information. The Δk [32] and Puechmaille’s [33] methods, as performed in StructureSelector [34] (http://Imme.ac.cn7StructureSelector/), were used to assess the most likely number of genetic clusters. Individual (q) and average (Q) proportions of membership in each of the inferred clusters were eventually derived from the multilocus genotype data of single falcon samples.

### Mitochondrial DNA

In order to investigate the genetic relationships of the sampled hierofalcons through variation in their mtDNAs, single sequences of partial CR (294 bp), 12S (263 bp) and cytB (428 bp) were amplified in all samples. Subsequently, sequences were concatenated in haplotypes of 985 bp in total length, edited and aligned using Geneious Prime (https://www.geneious.com). To compare broader geographic ranges for our falcon taxa, as well as to assess their relationships with unsampled *Falco* (sub)species, 47 already published CR sequences were downloaded from GenBank (S2 Table) and added to our dataset of single CR sequences in a second, shorter mtDNA alignment of 295 bp in length. To examine the relationships and distribution of our concatenated haplotypes, a median-joining network [35] was constructed using the software PopART [36]. Separately, a network was also derived using a shorter alignment consisting of our and online CR-based haplotypes. Nucleotide diversity (π) for concatenated haplotypes and single CR sequences was computed using DnaSP 6.0 [37].

## Results

### Coding genes and STRs

Of the 115 samples collected from *F*. *biarmicus* and *F*. *cherrug*, 3 museum samples actually showed mitochondrial haplotypes from peregrine falcon (*F*. *peregrinus*, see below) and were not analysed. Furthermore, 11 additional taxidermied specimens yielded unusable partial profiles and were discarded. Finally, genotypes at nuclear genes were obtained for a total of 101 hierofalcon samples (*F*. *b*. *feldeggii*, n = 55; *F*. *b*. *tanypterus*, n = 14, *F*. *b*. *erlangeri*, n = 10; *F*. *cherrug*, n = 22).

The reconstruction of haplotypes performed by PHASE was used to derive allelic states in the coding genes. Results from computations revealed five alleles for BDNF (4 segregating sites), 10 alleles for Cmos (6 segregating sites) and, surprisingly, only 4 alleles for MHC (5 segregating sites) across all genotyped samples. The polymorphic sites were all biallelic, and no positions with three or four segregating nucleotides were detected. A maximum of four polymorphic positions were found per individual in each gene. Only one unphased haplotype in the gene *Cmos*, found as unique polymorphism in *F*. *b*. *erlangeri*, was ambiguously reconstructed (phase probabilities < 0.90), and remained unresolved as missing allele. When sequences of real and reconstructed alleles were checked against the reference genome of *F*. *cherrug*, no evidence for pseudogenes was detected, nor was there any inconsistency with online homologous MHC sequences from other falcons [8, 12]. Allele 4, however, was found as a new highly frequent variant in *F*. *b*. *erlangeri* and *F*. *b*. *tanypterus* (S3 Table). Overall, allele variation was largely shared in all falcons, and only a few private alleles were found at low frequencies (ranging from 0.0094 at Cmos in *F*. *b*. *feldeggii* to 0.1875 at MHC in *F*. *b*. *erlangeri*) in each taxon (S3 Table).

All 12 STR loci were polymorphic in our hierofalcon taxa, showing from three (in FP89 and FP5) to eleven (in FP82-2) alleles per locus (S3 Table). Significant genotyping errors were not found by MICRO-CHECKER, nor was evidence for null alleles provided at any locus. Private alleles with frequency higher than 10% were rare in all taxa, two being found in *F*. *cherrug*, one each in *F*. *b*. *erlangeri* and *F*. *b*. *feldeggii*, and none in *F*. *b*. *tanypterus*. Significant linkage disequilibrium (p < 0.001) involved five locus-by-locus comparisons (FP13 x FP89, AGE-5 x FP107, FALVES-03 x FP107, AGE-5 x FP82-2, FP82-2 x FP347) and was found exclusively in *F*. *b*. *feldeggii*. Five is equal to the number of significant comparisons expected by chance based on 105 possible pairwise comparisons. Moreover, if LE is needed for powerful downstream analyses, minor violations do not prevent from identifying discrete clusters of close genotypes [38]. Consequently, for our purposes, no locus was excluded from subsequent computations. Finally, downstream data analyses were performed on a total of 101 falcon samples based on ultimate 15-locus genotypes that encompassed 12 STR loci and 3 coding genes, with only a minor fraction of missing values (4% of unsampled or unresolved alleles).

**Fig 2:**
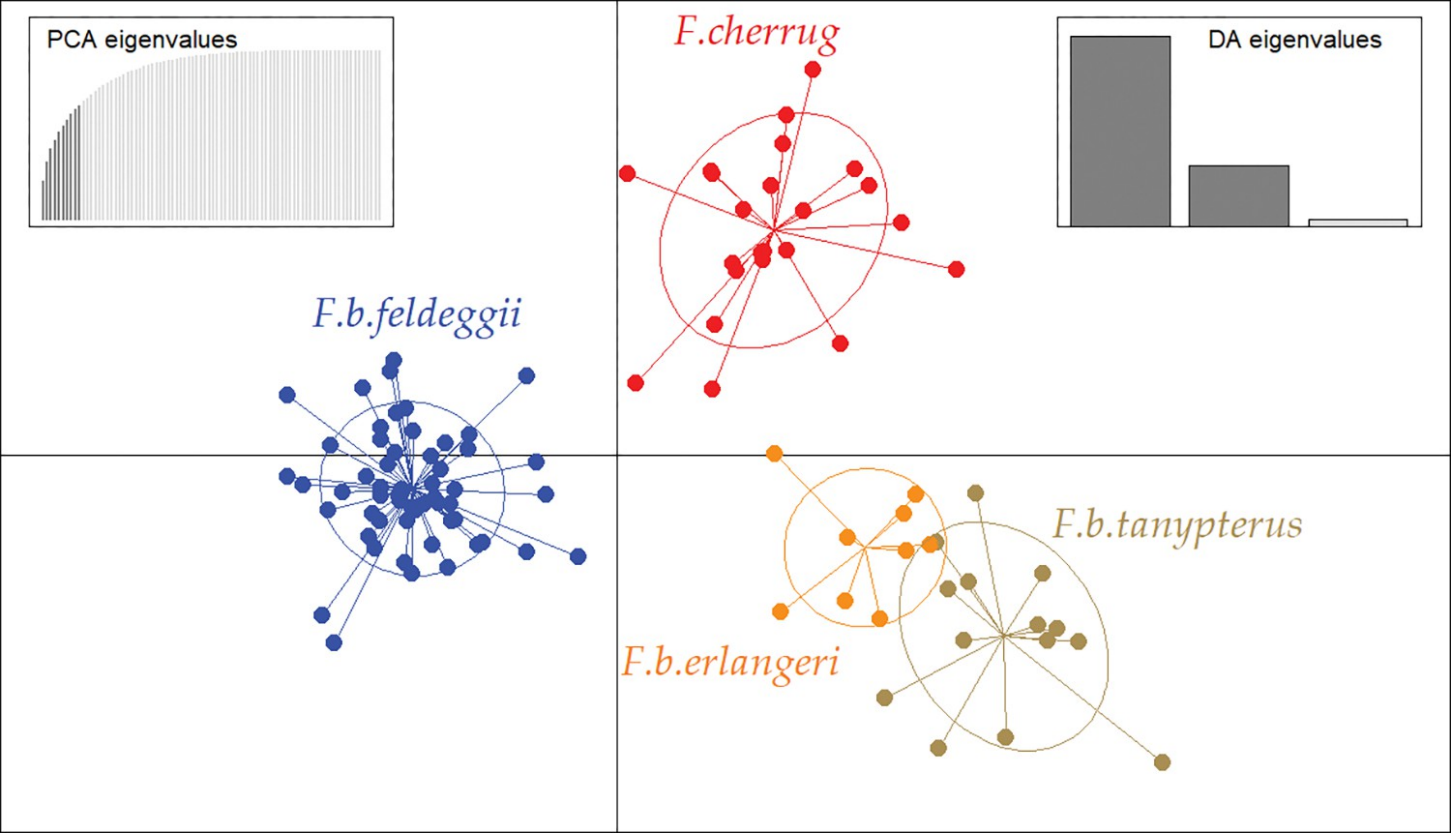
DAPC scatterplot based on a 15-locus panel describing the genetic variability in the sampled falcon taxa, identified by first (X-axis) and second (Y-axis) principal components. The graph features single individuals as dots and groups as inertia ellipses.

The overall genetic variability in the dataset was qualitatively described at the population level using DAPC (Fig 2). This multivariate approach uses principal components to summarise the original genetic data into uncorrelated discrete groups, and subsequently applies discriminant analysis to maximise the among-group variation. We entered four pre-defined populations into the computations, which corresponded to their specific (*F*. *cherrug*) and subspecific status (*F*. *b*. *feldeggii*, *F*. *b*. *erlangeri*, *F*. *b*. *tanypterus*) in accordance with taxonomy. Cross-validation procedure suggested that ten principal components (PC) were retained in the analysis as a proxy for optimal description of the original data and highest predictive capacity. The first two PCs best summarised most of the allelic diversity, and defined four clusters of genetically close individuals. The first PC identified the three *F*. *biarmicus* subspecies as separate clusters, with *feldeggii* being highly distinct. *F*. *b*. *erlangeri* and *F*. *b*. *tanypterus*, however, were not completely distinct clusters, showing some degree of overlapping and pointing to possible admixture. In particular, according to the individual membership probabilities provided by DAPC for our a-priori groups, one sample of *F*. *b*. *tanypterus* was actually assigned to the *F*. *b*. *erlangeri* cluster. The second PC clearly separated samples of saker falcon *F*. *cherrug* as a further discrete and distant cluster.

**Fig 3:**
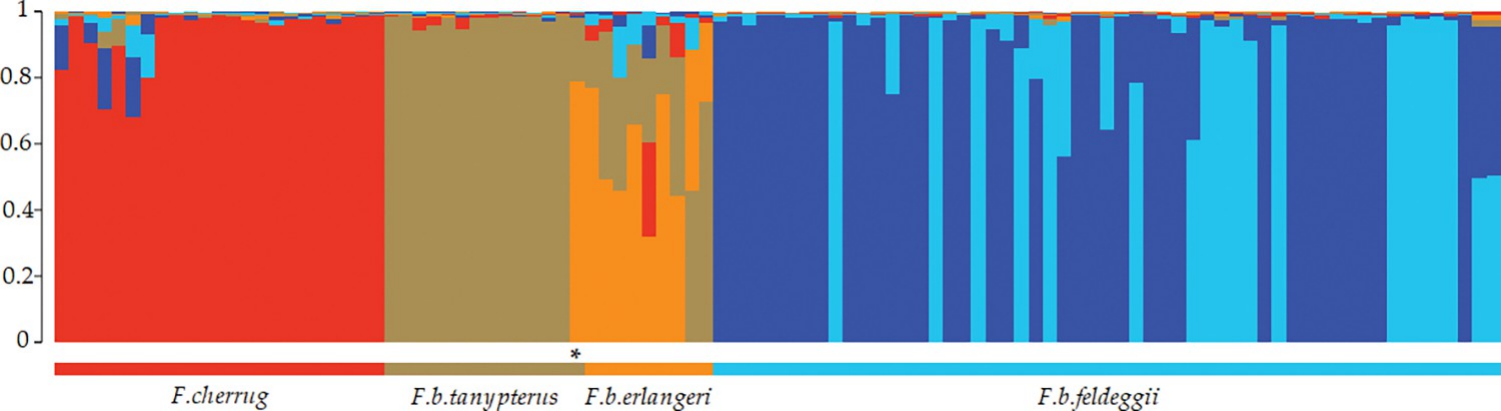
Bar plotting of the results from a Bayesian analysis conducted with STRUCTURE, showing five genetic clusters (K = 5). Each vertical bar represents one individual and the length of the different coloured sections is proportional to membership (q-values) in the inferred genetic clusters. Membership of *F*. *b*. *feldeggii* is distributed into two private clusters (see also Table 4). The star indicates one specimen of *tanypterus* that fell into the *erlangeri* group, as also revealed by DAPC. The bar plot was drawn using STRUCTURE PLOT [39] (http://omicsspeaks.com/strplot2/).

Single multilocus genotypes were used to assess the probability of their ancestry in one (or more) genetic clusters with the STRUCTURE Bayesian analysis for K ranging from 2 to 8. Both the Evanno Δk and Puechmaille’s method identified K = 5 as the most probable value for the number of inferred clusters. All lanners *F*. *b*. *feldeggii* were probabilistically assigned to two clusters (III and V) with a total mean Q = 0.988 (Table 4). Falcons from *F*. *b*. *tanypterus* and *F*. *cherrug* fell into two alternative clusters (II and I) showing Q values of 0.920 and 0.933, respectively. On the contrary, significant admixture was found in *F*. *b*. *erlangeri* (Fig 3, Table 4), where all samples, while falling into their own cluster (IV) with Q = 0.507, shared a non-negligible amount of ancestry with *F*. *b*. *tanypterus* (individual q values ranging from 0.143 to 0.726). Additionally, four of them showed also some admixture with *F*. *b*. *feldeggii* (q from 0.092 to 0.154), and two additional individuals with *F*. *cherrug* (q = 0.104 and 0.285). One sample of *F*. *b*. *tanypterus* fell into its own cluster (II) with only a small q-value (0.195), while most of its ancestry was shared with *F*. *b*. *erlangeri* (q = 0.788), as already suggested by DAPC. Conversely, one *F*. *b*. *erlangeri* shared ancestry in the *tanypterus* cluster (II) with high membership value (q = 0.722, Fig 3). The majority of saker falcons *F*. *cherrug* fell into cluster I; few individuals, however, showed from low to moderate membership to the *F*. *b*. *feldeggii* clusters (q values from 0.086 to 0.279). On the contrary, no shared ancestry with other falcon taxa was found in the *feldeggii* individuals, which showed values of membership to their clusters (III and V) all higher than or equal to 0.975.

**Table 4:** Values of average proportion of membership (Q) of falcon samples in the clusters inferred by STRUCTURE for K = 5.

	Cluster I	Cluster II	Cluster III	Cluster IV	Cluster V
*F*. *b*. *feldeggii*	0.005	0.004	0.336	0.004	0.652
*F*. *b*. *tanypterus*	0.011	0.920	0.004	0.061	0.005
*F*. *b*. *erlangeri*	0.061	0.360	0.051	0.507	0.022
*F*. *cherrug*	0.933	0.011	0.018	0.007	0.032

### Mitochondrial DNA

Amplifications of the mitochondrial marker sequences were performed in all collected samples. Seven taxidermied specimens, however, yielded no amplicons (one *F*. *b*. *feldeggii* from Dalmatia, dated 1847, two putative *F*. *b*. *erlangeri* from the museum in Malta, dating back to the 1940s, two *feldeggii* from the museum in Chieti, collected around the mid-1970s, and two *F*. *cherrug* stuffed at the Vienna Museum in 1975 and 2004). Furthermore, three specimens from the museums in Vienna and Chieti were misclassified as one *cherrug* and two *F*. *b*. *erlangeri*, respectively, while being actually peregrine falcons (*F*. *peregrinus*) based on their mtDNA. Finally, single sequences of 12S, cytB and the CR were obtained for a total of 105 falcon samples (*F*. *b*. *feldeggii*, N = 58; *F*. *b*. *tanypterus*, N = 14, *F*. *b*. *erlangeri*, N = 10; *F*. *cherrug*, N = 23).

Two haplotypes were found at 12S (263 bp) and four at cytB (428 bp), while seven haplotypes were identified at the CR (294 bp). Sequences have been deposited in GenBank, with accession numbers listed in S1 Table. The three fragments were concatenated to obtain sequences of 985 bp in total length. Eight concatenated haplotypes were identified in the falcon taxa (Table 1, S1 Table), based on 12 polymorphic sites, 5 of which were found in the CR, 5 in cytB and 2 in 12S. Except for one transversion in the 12S sequence, all point mutations were transitions. Haplotypes showed moderate differentiation, with nucleotide diversity (π) ranging from 0.00102 to 0.01218. Due to small sample sizes for most of the taxa studied, no inference was derived based on the frequency distribution of haplotypes. In 58 *feldeggii* individuals, however, only the concatenated haplotypes H3 and H6 were present, with frequencies of 0.273 and 0.727, respectively.

**Fig 4:**
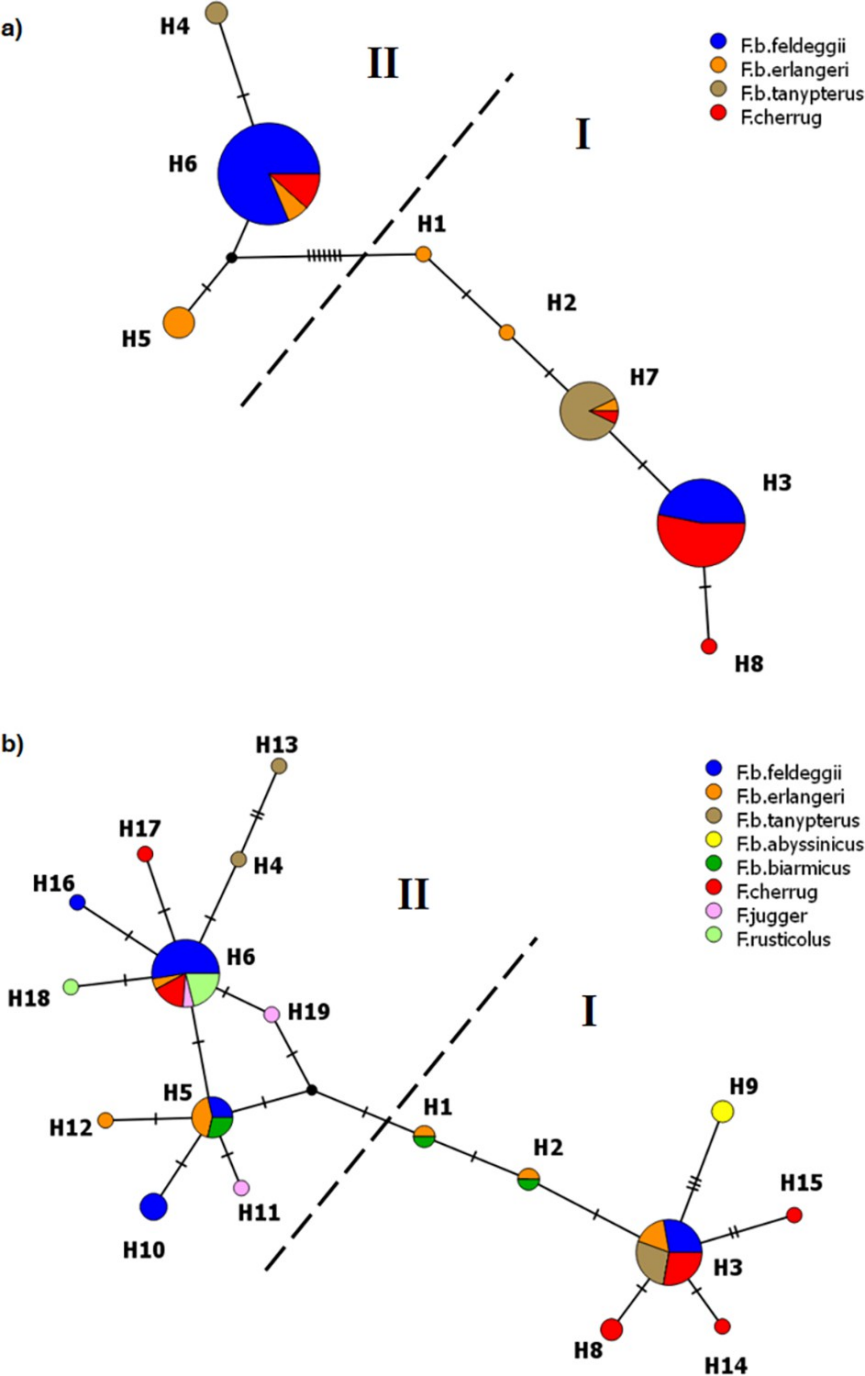
Median-joining networks based on the mitochondrial haplotypes of *Falco* taxa. Networks obtained using: a) a 985-bp alignment of concatenated sequences from our samples of *F*. *biarmicus* ssp. and *F*. *cherrug*; b) a trimmed 295-bp CR alignment including our and published sequences from several falcon (sub)species. Circles represent single haplotypes; the size of the circles is proportional to the haplotype frequency; colours represent the proportion of individuals from each (sub)species; ticks on connecting lines indicate mutational steps between haplotypes; small black nodes mark inferred (unsampled) sequences. Hatched lines separate two major haplogroups (I and II).

The genealogical relationships of concatenated haplotypes were inferred by a median-joining network, which clearly depicted two mixed mitochondrial haplogroups (I and II, Fig 4A), separated by a π-value of 0.00968. In comparison, nucleotide diversity within either haplogroup was smaller than nearly one order of magnitude (0.00203, sd 0.00047 and 0.00203, sd 0.00071 in haplogroups I and II, respectively). It is noteworthy that sample-by-sample inspection in *F*. *b*. *feldeggii* did not reveal any relationship between the distribution of specimens in the mitochondrial haplogroups and the membership clusters inferred by STRUCTURE (Fig 3, Table 4).

Sequences of the CR are the most represented online for the mitochondrial DNA in the genus *Falco*, therefore we aligned our seven single CR haplotypes with published sequences from eight hierofalcon (sub)species (S3 Table) in order to reconstruct their genetic relationships by including more genetic variability and a higher number of taxa. The network derived using a trimmed 295-bp alignment (Fig 4B) confirmed both our findings from concatenated haplotypes and the results from the literature, i.e. the existence of two distinct non-monophyletic mtDNA lineages, that were both present in different taxa and the same taxon from different geographic locations. Compared to our previous network based on concatenated haplotypes, the inclusion of greater variability led to an increase in nucleotide diversity within haplogroups (0.00939, sd 0.00191 and 0.00841, sd 0.00122 in I and II, respectively) and, proportionally, shorter distance between haplogroups (0.01882).

## Discussion

Efforts to protect wild taxa are highly endorsed, and conservation projects gain priority and funding attention, when their (phylo)genetic distinctiveness is assessed and taxonomic recognition is supported by the occurrence of unique hereditary characters. In the case of hierofalcons, this holds particularly true, and genetic studies face a twofold challenge: some of the extant (sub)species are rare, up to being to the brink of extinction, and many of them are genetically closely related, due to their evolutionary recent common ancestor [3]. This can make their diagnosability rather tricky. That said, however, the joint use of multiple neutral STR markers and coding genes under selection, as adopted in this study, proved a successful approach to differentiate our falcon taxa through non-trivial genetic differences [40] combined with statistical assigning procedures. In this framework, based on the allele number and frequency, coding genes proved less variable than neutral loci, as expected from lack of functional constrains for the latter and different mutational and evolutionary mechanisms. The second exon of the MHC class II B gene, in particular, showed unusually low variability, a scenario of genetic impoverishment already described in large falcons and confirmed in this study by an increased number of individuals and taxa than previously investigated [8, 12]. Natural selection mediated by particularly abundant and/or harmful pathogens could have driven the depletion of MHC variation via directional selection in the common ancestor of extant falcons. Compensatory mechanisms by other immune genes cannot be ruled out either [8, 12].

Partly disagreeing with earlier studies that indicated “strong genetic identity” for the three Palearctic subspecies of *F*. *biarmicus* [9], our results revealed that *F*. *b*. *feldeggii*, in particular, exhibited a nuclear gene pool that was highly distinct from the other subspecies of *F*. *biarmicus*, as well as from the saker falcon *F*. *cherrug*, revealing strong geographic signature and supporting the current taxonomy. Noteworthy, this result was obtained when allele frequency data were analysed either using an underlying genetic model, as in STRUCTURE, or without any model, as in DAPC. At the individual and population level, respectively, both approaches showed that no single *F*. *b*. *feldeggii* was admixed, i.e. their ancestry was not shared with any other falcon studied. Lanners from Sicily and the Balkans presumably share the same gene pool as the peninsular population, although we could not verify this directly, as individuals from those areas are underrepresented in our sampling. However, Sarà et al. [9] reported no significant genetic differences between continental Italy, Sicily and the Balkan area, and suggested the existence of a metapopulation with natural gene flow that prevents their isolation.

Differently from the European lanner *F*. *b*. *feldeggii*, the subspecies *F*. *b*. *tanypterus* from Israel and *F*. *b*. *erlangeri* from North Africa shared part of their nuclear gene pools, suggesting that they may be geographic populations of the same subspecies, as previously proposed [4, 5]. At the individual level, one *tanypterus* shared most of its ancestry in the *erlangeri* gene pool, and, vice versa, one *erlangeri* showed highest membership to the gene pool of *tanypterus*, indicating either morphological misidentification or interbreeding for both birds. Overall, falcons from North Africa appear as the most genetically admixed: this area could be as a meeting point and occasional interbreeding between resident and dispersing lanner falcons in the Mediterranean basin.

Nuclear DNA allowed a clear distinction also of the saker falcon *F*. *cherrug*. However, few individuals (5 out of 23) showed from low to moderate membership to the gene pool of *F*. *b*. *feldeggii*. This slight admixture can be explained by the fact that most of our saker samples were collected in Austria and Slovakia, i.e. the westernmost part of the distribution and the area closest to the lanner’s range. In these areas, the saker falcon is reported as a non-resident and non-breeding, but rather as a vagrant visitor species (http://datazone.birdlife.org/). In Bulgaria, the Balkans and Israel the saker can also overwinter [1]. Rare and rather recent hybridisation [41] may explain the signature of limited gene flow that we have observed. It is noteworthy, however, that introgression is asymmetrical, in that it occurs from *F*. *feldeggii* into *F*. *cherrug*, and not vice versa. This finding, as well as the high homogeneity of the *feldeggii* gene pool, can be explained by the loss of genetic diversity as a consequence of the severe bottleneck that it experienced in the late 19th and early 20th century across the entire range [6, 7]. Hybridisation events with the saker falcon may have predated this dramatic population decline. Bottleneck effects, however, may have removed traces of its ancestry from the *feldeggii*’s gene pool. In conclusion, while accounting for some uncertainty regarding *F*. *b*. *tanypterus* and *F*. *b*. *erlangeri*, the grouping of samples based on taxonomy was substantially confirmed by our nuclear genetic data, which indicate, in particular, *F*. *b*. *feldeggii* as the most divergent subspecies of *F*. *biarmicus* in the Mediterranean area.

While nuclear genetic data basically supports current taxonomy, however this does not hold true with mtDNA. The network, based on our and online sequences, identified two different lineages, but clearly showed that no falcon taxon formed a monophyletic group, nor were any relationships found with their respective distribution areas: the haplotypes found in *F*. *cherrug* and subspecies of *F*. *biarmicus* from different Mediterranean sites were dispersed in both lineages, suggesting the lack of diagnostic power for mitochondrial markers in these birds. Overall, the haplotypes were poorly differentiated from each other, with a few haplotypes being largely widespread across taxa, while others occurring only rarely. These results fully agree with earlier findings in highlighting paraphyly for the *Falco* species and the mismatch between haplogroups and taxonomic entities [1, 3]. Furthermore, they point to the real difficulty of finding solid explanations for the current phylogeographic pattern obtained in these birds [1, 3], a picture that remains largely speculative.

The lanner is the most critically endangered hierofalcon in Europe. The species is worthy of high conservation priority especially in Italy [42], that hosts the most important population and a considerable portion of the world genetic stock of the *feldeggii* subspecies [43, 44]. Thus, a crucial role and great responsibility is assumed by this country for the conservation of the taxon at a global level [7]. To address the issue, with the purpose of increasing population size, or at least halting its decline, five-year conservation LIFE projects with EU funds were launched in 2010 in Sicily (LIFE ConRaSI) and in 2020 in central Italy (LIFE LANNER), with the latter including captive breeding and release of birds into the wild. One of the goals of the projects was to highlight the genetic distinctiveness of the lanner, in order to endorse and encourage, if ever needed, the efforts for its conservation. Our results showed that selected nuclear markers were capable of genetically distinguishing the European lanner both at the population and individual level. Although not supported by mitochondrial data (not surprisingly, however, [1, 3]), this genetic uniqueness, combined with the existence of distinctive non-genetic traits in morphology, distribution and ecology, strongly claims for the conservation argument and emphasises the need for urgent interventions. Without timely and effective actions, this bird risks disappearing forever in the wild, adding to the already long list of species that witness the loss of biodiversity.

The rarity of lanners and the conservation policy of “no impact” on the wild population, has led to rely on captive-bred instead of wild-sourced falcons for the recruitment of individuals in projects involving the release of birds into the wild (cfr. LIFE LANNER). In such a scenario, monitoring captive falcons is crucial. In this regard, molecular identification of single birds, individual membership to a specific gene pool (i.e. subspecies), assessment of parental pairs, and reconstruction of genetic relationships among generations are essential to select the individuals to be released in nature (Lorenzini et al. in prep.). In addition to assess genealogy and ancestry, our panel of nuclear markers proved a valuable tool to identify genetically admixed individuals. This application is much desired when occasional intra- or interspecific hybrids, either natural or anthropogenic, are to be identified for management, scientific and forensic purposes.

## Supporting information

S1 Table List of samples, origin, year of collection, single and concatenated mitochondrial haplotypes. The order of samples corresponds to the order of samples in the barplot (Fig 3 in the main text). GenBank accession numbers are indicated in parentheses.
(PDF)

S2 Table List of 47 mitochondrial CR sequences downloaded from GenBank and used to construct a median-joining network. H1-H8 indicate the haplotypes obtained in this study, corresponding to sequences previously published elsewhere with the accession numbers below.
(PDF)

S3 Table Allele frequencies at 12 STR loci and 3 nuclear coding genes in four hierofalcon taxa. Sample size in parenthesis.
(PDF)
