# Supplementary material for: Genetic distinctiveness of an endangered falcon: Implications for conservation in Europe
Source: PLoS One. 2023 Dec 20;18(12):e0295424. doi: 10.1371/journal.pone.0295424 (PMC10732417; doi:10.1371/journal.pone.0295424)
Supplement: S1 Table — The order of samples corresponds to the order of samples in the barplot (Fig 3 in the main text). GenBank accession numbers are indicated in parentheses. (PDF) [file pone.0295424.s001.pdf]

Genetic distinctiveness of an endangered falcon: implications for conservation in Europe

Lorenzo Attili[1,2], Luisa Garofalo[3] *, Giuseppe Puddu[4], Giampiero Tirone[4], Antonella Pizzarelli[1], Nicholas Barbara[5], Elisabeth Haring[6], Rita Lorenzini[1]

[1] Istituto Zooprofilattico Sperimentale del Lazio e della Toscana *M. Aleandri*, Centro di Referenza Nazionale per la Medicina Forense Veterinaria, Grosseto, Italy

[2] Department of Biology and Biotechnology "*C. Darwin*", Sapienza University of Rome, Italy

[3] Istituto Zooprofilattico Sperimentale del Lazio e della Toscana *M. Aleandri*, Rome, Italy

[4] Regione Lazio, Riserva Naturale Lago di Vico, Caprarola, Viterbo, Italy

[5] Birdlife Malta, Ta' Xbiex, Malta

[6] Natural History Museum Vienna, Wien, Austria

* Corresponding author: luisa.garofalo@izslt.it

Table S1. List of samples, origin, year of collection, single and concatenated mitochondrial haplotypes. The order of samples corresponds to the order of samples in the barplot (Fig. 3 in the main text). GenBank accession numbers are indicated in parentheses.

Taxon	ID	Origin	Year	12S	cytB	CR	Concatenated haplotype
Falco cherrug	NMW 60.160	Austria[c]	1953	LL6 (OR392673)	LL2 (OR333773)	H3 (OR333828)	H7
	NMW 95.235	Unknown[c]	2002	LL6	LL3 (OR333774)	H3	H3
	NMW 95.297	Unknown[c]	2009	LL6	LL3	H3	H3
	NMW 96.927	Austria[c]	2015	LL6	LL3	H3	H3
	NMW 98.546	Unknown[c]	2012	LL5 (OR392678)	LL1 (OR333772)	H6 (OR333834)	H6
	NHMW-DNAtis_25132	Austria[c]	Unknown	LL6	LL3	H3	H3
	NHMW-DNAtis_25184	Austria[c]	2003	LL5	LL1	H6	H6
	NHMW-DNAtis_25176	Austria[c]	2003	LL6	LL3	H3	H3
	NHMW-DNAtis_25187	Unknown[c]	2003	LL5	LL1	H6	H6
	NHMW-DNAtis_25134	Unknown[c]	1982	LL6	LL3	H3	H3
	NHMW-DNAtis_25139	Austria[c]	1984	LL6	LL3	H3	H3
	NHMW-DNAtis_25140	Austria[c]	1895	LL6	LL3	H3	H3
	NHMW-DNAtis_25136	Austria[c]	1929	LL5	LL1	H6	H6
	NMW 73.565	Austria[c]	1931	LL6	LL3	H3	H3
	NMW 75.152	Austria[c]	1977	LL6	LL3	H3	H3
	NMW 82.961	Unknown[c]	1989	LL6	LL3	H8 (OR333831)	H8
	NMW 82.962	Unknown[c]	1989	LL6	LL3	H3	H3
	NHMW-DNAtis_25059	Austria[c]	2003	LL6	LL3	H3	H3
	NHMW-DNAtis_25145	Slovakia[c]	2000	LL6	LL3	H3	H3
	NHMW-DNAtis_25250	Austria[c]	2003	LL6	LL3	H3	H3
	NHMW-DNAtis_25183	Austria[c]	2003	LL6	LL3	H3	H3
	NHMW-DNAtis_25159	Slovakia[c]	1980	LL6	LL3	H3	H3
	NHMW-DNAtis_25135	Austria[c]	1860	LL5	LL1	H6	H6
	NHMW-DNAtis_25142	Austria[c]	1960	*Falco peregrinus*			
	NHMW-DNAtis_25138	Austria[c]	1975	*na*	*na*	*na*	*na*
	NHMW-DNAtis_25203	Austria[c]	2004	*na*	*na*	*na*	*na*
Falco biarmicus tanypterus	HY321-R	Israel[e]	2021	LL6 (OM743842)	LL2 (OR344391)	H3 (OR333827)	H7
	G-26979	Israel[e]	2021	LL6	LL2	H3	H7

	HY303	Israel[e]	2021	LL6	LL2	H3	H7
	G-26986	Israel[e]	2021	LL5 (OR392676)	LL1 (OR344390)	H4 (OR333829)	H4
	G-26985	Israel[e]	2021	LL5	LL1	H4	H4
	G-26449	Israel[e]	2021	LL6	LL2	H3	H7
	GG-5938	Israel[e]	2021	LL6	LL2	H3	H7
	G-26444	Israel[e]	2021	LL6	LL2	H3	H7
	GG-5917	Israel[e]	2021	LL6	LL2	H3	H7
	G-26982	Israel[e]	2021	LL6	LL2	H3	H7
	G-26984	Israel[e]	2021	LL6	LL2	H3	H7
	G-26442	Israel[e]	2021	LL6	LL2	H3	H7
	G-26438	Israel[e]	2021	LL6	LL2	H3	H7
	G-26362	Israel[e]	2021	LL6	LL2	H3	H7
Falco biarmicus erlangeri	16057452-1	North Africa[e]	2016	LL5 (OR392677)	LL1 (OR344393)	H6 (OR333833)	H6
	16057452-2	North Africa[e]	2016	LL6 (OM743841)	LL2 (OR344392)	H1 (OR333823)	H1
	16057452-3	North Africa[e]	2016	LL6	LL2	H2 (OR333824)	H2
	NMNH-0096	Egypt[d]	Unknown	LL5	LL4 (OR333771)	H5 (OR333830)	H5
	NMNH-0428	Egypt[d]	Unknown	*Falco peregrinus*			
	NMNH-0429	Egypt[d]	Unknown	LL5	LL1	H6	H6
	NMNH-0838	Egypt[d]	Unknown	LL5	LL4	H5	H5
	NMNH-0880	Egypt[d]	Unknown	LL6	LL2	H3 (OR333826)	H7
	NMNH-0892	Egypt[d]	Unknown	LL5	LL4	H5	H5
	NMNH-0919	Egypt[d]	Unknown	LL5	LL4	H5	H5
	NMNH/ORN 2623	North Africa[d]	1973	LL5	LL1	H6	H6
	NMNH/ORN 3312	North Africa[d]	Unknown	*na*	*na*	*na*	*na*
	NMNH/ORN 0341	North Africa[d]	1983	*Falco peregrinus*			
	NMNH/ORN 2453	North Africa[d]	1940s	*na*	*na*	*na*	*na*
Falco biarmicus feldeggii	16057452-4	Peninsular Italy[e]	2005	LL6 (OR392674)	LL3 (OR469850)	H3 (OR333825)	H3
	FG0985	Peninsular Italy[e]	2008	LL5 (OR392675)	LL1 (OR344389)	H6 (OR333832)	H6
	FG0984	Peninsular Italy[e]	2008	LL5	LL1	H6	H6
	FG0989	Peninsular Italy[e]	2016	LL5	LL1	H6	H6
	FG0966	Peninsular Italy[e]	2016	LL5	LL1	H6	H6
	FG0982	Peninsular Italy[e]	2016	LL5	LL1	H6	H6
	FG0964	Peninsular Italy[e]	2016	LL5	LL1	H6	H6

	FG0980	Peninsular Italy[e]	2016	LL5	LL1	H6	H6
	FG0981	Peninsular Italy[e]	2016	LL5	LL1	H6	H6
	D BNA Z 04 404	Peninsular Italy[e]	2017	LL6	LL3	H3	H3
	10 BG 555	Peninsular Italy[e]	2017	LL6	LL3	H3	H3
	52FOIL11900A	Peninsular Italy[e]	2017	LL5	LL1	H6	H6
	EF136599	Peninsular Italy[e]	2017	LL5	LL1	H6	H6
	LS21/CF9002	Peninsular Italy[e]	2020	LL6	LL3	H3	H3
	21FOIIT20900A	Peninsular Italy[e]	2020	LL5	LL1	H6	H6
	22FOIIT20900A	Peninsular Italy[e]	2020	LL5	LL1	H6	H6
	23FOIIT20900A	Peninsular Italy[e]	2020	LL5	LL1	H6	H6
	06PPB15	Peninsular Italy[e]	2020	LL5	LL1	H6	H6
	ZG 12,0 090179	Peninsular Italy[e]	2020	LL5	LL1	H6	H6
	EF 11 65 36	Peninsular Italy[e]	2020	LL5	LL1	H6	H6
	02PPB2017	Peninsular Italy[e]	2017	LL5	LL1	H6	H6
	MCCI 4219	Peninsular Italy[a]	2013	LL5	LL1	H6	H6
	MCCI 4220	Sicily[a]	2012	LL5	LL1	H6	H6
	27ppb 2017	Peninsular Italy[e]	2017	LL5	LL1	H6	H6
	01LS	Peninsular Italy[e]	2020	LL6	LL3	H3	H3
	150.402	Peninsular Italy[e]	2020	LL5	LL1	H6	H6
	Nard U221 51	Peninsular Italy[e]	2020	LL5	LL1	H6	H6
	PZB008	Peninsular Italy[e]	2021	LL5	LL1	H6	H6
	55 FOI15 900A	Peninsular Italy[e]	2021	LL5	LL1	H6	H6
	EF116523	Peninsular Italy[e]	2021	LL5	LL1	H6	H6
	2FOI L16 900A	Peninsular Italy[e]	2021	LL5	LL1	H6	H6
	06 LS	Peninsular Italy[e]	2021	LL6	LL3	H3	H3
	4 FOI19 900A	Peninsular Italy[e]	2021	LL5	LL1	H6	H6
	21061304	Peninsular Italy[e]	2021	LL6	LL3	H3	H3
	9781101082116145	Peninsular Italy[e]	2021	LL6	LL3	H3	H3
	20/0332	Spain[e]	2021	LL5	LL1	H6	H6
	20/0333	Spain[e]	2021	LL5	LL1	H6	H6
	12BG678	Peninsular Italy[e]	2021	LL6	LL3	H3	H3
	13, ODBNANG10024	Peninsular Italy[e]	2021	LL5	LL1	H6	H6
	12, ODBNADFOG09003	Peninsular Italy[e]	2021	LL5	LL1	H6	H6
	ZG12, 0/09/183	Peninsular Italy[e]	2021	LL5	LL1	H6	H6
	23PPB2012	Peninsular Italy[e]	2021	LL5	LL1	H6	H6

	22FOIIT21900A12,5	Peninsular Italy[e]	2021	LL5	LL1	H6	H6
	21/7464	Spain[e]	2022	LL6	LL3	H3	H3
	21/7465	Spain[e]	2022	LL6	LL3	H3	H3
	21/7466	Spain[e]	2022	LL6	LL3	H3	H3
	21/7467	Spain[e]	2022	LL6	LL3	H3	H3
	21/7469	Spain[e]	2022	LL6	LL3	H3	H3
	21/7470	Spain[e]	2022	LL6	LL3	H3	H3
	PPB 6-17-2011	Peninsular Italy[e]	2020	LL5	LL1	H6	H6
	PPB 6-12-2011	Peninsular Italy[e]	2020	LL5	LL1	H6	H6
	CN 26	Peninsular Italy[e]	2020	LL5	LL1	H6	H6
	CN 27	Peninsular Italy[e]	2020	LL5	LL1	H6	H6
	CN 28	Peninsular Italy[e]	2020	LL5	LL1	H6	H6
	IT/CE/2003/PE/00020_A3	Peninsular Italy[b]	~1970	*na*	*na*	*na*	*na*
	IT/CE/2003/PE/00020_A1	Peninsular Italy[b]	Unknown	LL5	LL1	H6	H6
	IT/CE/2003/PE/00020_A4	Peninsular Italy[b]	Unknown	*na*	*na*	*na*	*na*
	IT/CE/2003/PE/00020_A2	Peninsular Italy[b]	Unknown	LL5	LL1	H6	H6
	22072190	Peninsular Italy[e]	2022	LL5	LL1	H6	H6
	NMW 37.740	Dalmatia[c]	1847	*na*	*na*	*na*	*na*
	NHMW-DNAtis_25309	Peninsular Italy[c]	1987	LL5	LL1	H6	H6

[a] Carmagnola Natural History Museum (Torino, Italy); [b] Museum of the University "G. D'Annunzio" (Chieti, Italy); [c] Natural History Museum Vienna (Wien, Austria); [d] National Museum of Natural History (Mdina, Malta); [e] Field.
