# Supplementary material for: Genetic distinctiveness of an endangered falcon: Implications for conservation in Europe
Source: PLoS One. 2023 Dec 20;18(12):e0295424. doi: 10.1371/journal.pone.0295424 (PMC10732417; doi:10.1371/journal.pone.0295424)
Supplement: S2 Table — H1-H8 indicate the haplotypes obtained in this study, corresponding to sequences previously published elsewhere with the accession numbers below. (PDF) [file pone.0295424.s002.pdf]

Genetic distinctiveness of an endangered falcon: implications for conservation in Europe

Lorenzo Attili[1,2], Luisa Garofalo[3] *, Giuseppe Puddu[4], Giampiero Tirone[4], Antonella Pizzarelli[1], Nicholas Barbara[5], Elisabeth Haring[6], Rita Lorenzini[1]

[1] Istituto Zooprofilattico Sperimentale del Lazio e della Toscana *M. Aleandri*, Centro di Referenza Nazionale per la Medicina Forense Veterinaria, Grosseto, Italy

[2] Department of Biology and Biotechnology "*C. Darwin*", Sapienza University of Rome, Italy

[3] Istituto Zooprofilattico Sperimentale del Lazio e della Toscana *M. Aleandri*, Rome, Italy

[4] Regione Lazio, Riserva Naturale Lago di Vico, Caprarola, Viterbo, Italy

[5] Birdlife Malta, Ta' Xbiex, Malta

[6] Natural History Museum Vienna,Wien, Austria

* Corresponding author: luisa.garofalo@izslt.it

Table S2. List of 47 mitochondrial CR sequences downloaded from GenBank and used in PopArt to construct a median-joining network. **H1-H8** indicate the haplotypes obtained in this study, corresponding to sequences previously published elsewhere with the accession numbers below.

**Haplotype**	**Taxon**	**Accession number**
**H1**	*Falco biarmicus biarmicus*	DQ144165
**H2**	*F. b. biarmicus*	DQ144139
**H3**	*Falco cherrug*	DQ144166<br>DQ144167<br>DQ144168<br>DQ144169<br>DQ144177
**H4**	*Falco biarmicus tanypterus*	DQ144138
**H5**	*Falco biarmicus feldeggii*<br><br><br>*F. b. biarmicus*	MK790096<br>DQ144156<br>DQ144157<br>DQ144146
**H6**	*F. b. feldeggii*<br><br><br><br><br><br><br><br><br><br>*F. cherrug*<br><br><br>*Falco jugger*<br>*Falco rusticolus*	MK790074<br>MK790075<br>MK790084<br>MK790085<br>MK790086<br>MK790088<br>MK790092<br>MK790093<br>MK790094<br>DQ144160<br>DQ144172<br>DQ144173<br>DQ144179<br>DQ144181<br>DQ144190<br>DQ144191<br>DQ144193<br>DQ144194<br>DQ144196<br>DQ144197
**H8**	*F. cherrug*	EF126937
H9	*Falco biarmicus abyssinicus*	DQ144142<br>DQ144162
H10	*F. b .feldeggii*	MK790077<br>MK790078<br>MK790080
H11	*F. jugger*	DQ144180
H12	*Falco biarmicus erlangeri*	DQ144159
H13	*F. b. tanypterus*	DQ144137
H14	*F. cherrug*	DQ144171
H15	*F. cherrug*	DQ144178
H16	*F. b. feldeggii*	DQ144161
H17	*F. cherrug*	DQ144170
H18	*F. rusticolus*	DQ144192
H19	*F. jugger*	DQ144183
