# Supplementary material for: Genetic distinctiveness of an endangered falcon: Implications for conservation in Europe
Source: PLoS One. 2023 Dec 20;18(12):e0295424. doi: 10.1371/journal.pone.0295424 (PMC10732417; doi:10.1371/journal.pone.0295424)
Supplement: S3 Table — Sample size in parenthesis. (PDF) [file pone.0295424.s003.pdf]

Genetic distinctiveness of an endangered falcon: implications for conservation in Europe

Lorenzo Attili[1,2], Luisa Garofalo[3] *, Giuseppe Puddu[4], Giampiero Tirone[4], Antonella Pizzarelli[1], Nicholas Barbara[5], Elisabeth Haring[6], Rita Lorenzini[1]

[1] Istituto Zooprofilattico Sperimentale del Lazio e della Toscana *M. Aleandri*, Centro di Referenza Nazionale per la Medicina Forense Veterinaria, Grosseto, Italy

[2] Department of Biology and Biotechnology "*C. Darwin*", Sapienza University of Rome, Italy

[3] Istituto Zooprofilattico Sperimentale del Lazio e della Toscana *M. Aleandri*, Rome, Italy

[4] Regione Lazio, Riserva Naturale Lago di Vico, Caprarola, Viterbo, Italy

[5] Birdlife Malta, Ta' Xbiex, Malta

[6] Natural History Museum Vienna,Wien, Austria

* Corresponding author: luisa.garofalo@izslt.it

Table S3. Allele frequencies at 12 STR loci and 3 nuclear coding genes in four hierofalcon taxa. Sample size in parenthesis.

**Locus**	**Allele**	***F. b. erlangeri* *(10)***	***F. b. feldeggii* *(55)***	***F. b. tanypterus* *(14)***	***F. cherrug* *(22)***
AGE-5	153	0.0000	0.0000	0.0000	0.0208
	159	0.1667	0.0364	0.1071	0.0417
	161	0.0000	0.0182	0.0000	0.0000
	163	0.3333	0.1636	0.1786	0.4375
	165	0.3333	0.2273	0.7143	0.4167
	169	0.1667	0.5545	0.0000	0.0833
FALVES-03	159	0.0000	0.0000	0.0714	0.0000
	162	0.1667	0.0000	0.4643	0.2727
	165	0.1667	0.0000	0.0000	0.0455
	168	0.0000	0.1111	0.1429	0.5000
	171	0.2778	0.0000	0.0000	0.0909
	174	0.0556	0.5833	0.1786	0.0000
	177	0.1111	0.3056	0.0000	0.0909
	180	0.0556	0.0000	0.0000	0.0000
	186	0.0556	0.0000	0.1429	0.0000
	189	0.1111	0.0000	0.0000	0.0000
FP13	94	0.0556	0.0000	0.0000	0.0000
	96	0.4444	0.0182	0.0000	0.0217
	98	0.1667	0.8091	0.3929	0.7391
	100	0.2778	0.0818	0.6071	0.2174
	102	0.0556	0.0909	0.0000	0.0217
FP54	100	0.0625	0.1204	0.0000	0.0500
	102	0.1250	0.1389	0.0000	0.0000
	104	0.2500	0.1019	0.1071	0.4000
	106	0.4375	0.0093	0.6071	0.3250
	108	0.0625	0.6111	0.0000	0.1250
	110	0.0000	0.0000	0.0000	0.0750
	112	0.0625	0.0000	0.2857	0.0000
	118	0.0000	0.0185	0.0000	0.0000
	120	0.0000	0.0000	0.0000	0.0250
FP79-1	140	0.2778	0.0000	0.0714	0.0000
	142	0.5000	0.6000	0.1071	0.6739
	144	0.2222	0.4000	0.8214	0.2826
	146	0.0000	0.0000	0.0000	0.0435
FP89	118	0.0000	0.0000	0.0000	0.0208
	120	0.2778	0.8364	0.1786	0.8333
	122	0.7222	0.1636	0.8214	0.1458
FALVES-31	119	0.0000	0.0000	0.0000	0.0909

	123	0.1667	0.2909	0.1071	0.0682
	127	0.5556	0.4818	0.2857	0.6591
	131	0.2222	0.0000	0.3571	0.0455
	135	0.0000	0.1091	0.0000	0.0455
	139	0.0000	0.1182	0.0000	0.0000
	143	0.0556	0.0000	0.2500	0.0227
	153	0.0000	0.0000	0.0000	0.0682
FP107	191	0.0000	0.1038	0.0000	0.0217
	201	0.0000	0.0283	0.0000	0.0870
	205	0.6111	0.6226	0.8214	0.1304
	206	0.1111	0.0000	0.0000	0.1739
	207	0.2222	0.2453	0.1786	0.5652
	209	0.0556	0.0000	0.0000	0.0217
FP31	144	0.0000	0.0000	0.0000	0.0217
	146	0.5000	0.1000	0.2500	0.7826
	148	0.1111	0.0727	0.1429	0.0000
	150	0.0556	0.0000	0.0000	0.0000
	152	0.0556	0.8273	0.2143	0.1522
	154	0.0556	0.0000	0.1071	0.0217
	156	0.2222	0.0000	0.2857	0.0217
FP82-2	131	0.0000	0.0000	0.0000	0.0625
	133	0.3889	0.2273	0.7143	0.0833
	135	0.1667	0.0727	0.0000	0.1667
	137	0.0556	0.0000	0.0714	0.1875
	139	0.0556	0.0000	0.0357	0.1250
	142	0.0556	0.0000	0.1786	0.0000
	145	0.0000	0.0909	0.0000	0.0000
	147	0.1667	0.3909	0.0000	0.2083
	149	0.1111	0.2182	0.0000	0.1042
	151	0.0000	0.0000	0.0000	0.0417
	153	0.0000	0.0000	0.0000	0.0208
FP347	130	0.0000	0.0000	0.0000	0.0208
	132	0.0000	0.0091	0.0000	0.0000
	134	0.0000	0.0000	0.0000	0.1458
	136	0.4444	0.1818	0.3929	0.4167
	138	0.1111	0.2364	0.4286	0.2500
	140	0.3333	0.2727	0.1786	0.0208
	148	0.0556	0.0000	0.0000	0.0208
	150	0.0000	0.0000	0.0000	0.0208
	152	0.0556	0.3000	0.0000	0.0000
	154	0.0000	0.0000	0.0000	0.1042
FP5	97	0.0000	0.0000	0.1071	0.0208
	101	0.6111	0.0273	0.5714	0.0000
	103	0.3889	0.9727	0.3214	0.9792
BDNF	1	0.0000	0.7600	0.0000	0.0357

	2	0.1000	0.1900	0.0769	0.0357
	3	0.4000	0.0500	0.7692	0.8929
	4	0.0000	0.0000	0.0000	0.0357
	5	0.5000	0.0000	0.1538	0.0000
CMOS	1	0.0000	0.0094	0.0000	0.0000
	2	0.0000	0.1132	0.0000	0.0000
	3	0.1667	0.4811	0.4615	0.0909
	4	0.0833	0.0000	0.0000	0.0000
	5	0.3333	0.1698	0.3077	0.0455
	6	0.0833	0.0000	0.0000	0.0000
	7	0.0000	0.0000	0.0385	0.0000
	8	0.1667	0.0000	0.0000	0.2727
	9	0.1667	0.2264	0.1923	0.5000
	10	0.0000	0.0000	0.0000	0.0909
MHC	1	0.1875	0.0566	0.5357	0.8611
	2	0.1875	0.0000	0.0000	0.0000
	3	0.2500	0.9434	0.0357	0.1389
	4	0.3750	0.0000	0.4286	0.0000
